# Impact of Laboratory-Accelerated Aging Methods to Study Alkali–Silica Reaction and Reinforcement Corrosion on the Properties of Concrete

**DOI:** 10.3390/ma13153273

**Published:** 2020-07-23

**Authors:** Arezou Attar, Bora Gencturk, Hadi Aryan, Jianqiang Wei

**Affiliations:** 1 Structural Engineer III, Mueser Rutledge Consulting Engineers, New York, NY 10122, USA; attar.arezo@gmail.com; 2 Sonny Astani Department of Civil and Environmental Engineering, University of Southern California, 3620 S. Vermont Avenue, KAP 210, Los Angeles, CA 90089-2531, USA; haryan@usc.edu; 3 Department of Civil and Environmental Engineering, University of Massachusetts, Lowell, MA 01854, USA; Jianqiang_wei@uml.edu

**Keywords:** steel reinforced concrete, mild steel, alkali–silica reactivity, rebar corrosion, accelerated aging, cracking

## Abstract

This study focuses on two separate investigations of the main aging mechanisms: alkali–silica reactivity (ASR) and the corrosion of reinforcing steel (rebar) concrete, both of which may result in a premature failure to meet the serviceability or strength requirements of a concrete structure. However, these processes occur very slowly, spanning decades. The impact of direct chemical additives to fresh concrete to accelerate ASR and the corrosion of reinforcing steel on the fresh and hardened properties of the ensuing material are investigated to inform the potential use of chemicals in large-scale studies. The deterioration of reinforced concrete (RC) is determined by means of expansion, cracking, bulk diffusivity and surface resistivity measurements, and compressive, split tensile and flexural strength tests. The results indicate that the addition of sodium hydroxide and calcium chloride can effectively accelerate the crack formation and propagation in concrete due to ASR and the corrosion of rebar, respectively. The ASR-induced cracks maintained a constant crack width from 0.05 mm to 0.1 mm over the measurement period regardless of the intensity of aging acceleration. Adding 4% chloride by weight of cement for accelerating rebar corrosion resulted in an average crack that was 82% larger than in the case of ASR accelerated with the addition of sodium hydroxide. The addition of alkali resulted in an increase in early-age (7-day) strength. At a total alkali loading of 2.98 kg/m^3^, 3.84 kg/m^3^ and 5.57 kg/m^3^, the 28-day compressive strength of concrete decreased by 3%, 10% and 24%, respectively. Similarly, a higher early-age strength and a lower later-age strength was observed for the concrete in the presence of corrosive calcium chloride. The results from this research are expected to inform future studies on the long-term performance of RC structures under accelerated ASR and corrosion.

## 1. Introduction

Aging in reinforced concrete (RC) structures occurs due to a combination of physical [1,2,3], thermal [4,5], chemical [6,7,8,9] and mechanical [10] effects. The aging mechanisms may include cracking due to temperature variations [11], creep and shrinkage [12], the alkali–silica reactivity (ASR) of concrete [13,14,15] and the corrosion of reinforcing bars (rebar) [16,17]. These effects may result in lower mechanical resistance and failure to meet serviceability or strength criteria in RC structures.

ASR is a pathogenic reaction in cement composites and is known as a major degradation mechanism in concrete structures [14]. Pesavento et al. [18] explained ASR as the reaction of the hydroxyl ion (OH^−^) of the alkalis (sodium and potassium) from hydraulic cement or other sources with the silanol groups (Si-O), and the breaking of the siloxane bonds of the silicon atom from the lattice [19], forming a hydrophilic alkali–silica gel consisting of silica, alkalis, water and other ions [18]. The swelling of this gel generates stresses and osmotic pressure, and may result in in the cracking and failure of the concrete [20]. The swelling is known to occur at a relative humidity (RH) higher than 80% [21]. To study ASR in a short period of time in a laboratory environment, two methods have commonly been used: (i) immersion in an alkaline solution [22] (simultaneously using high-alkali content cement in some references [23,24]); and (ii) adding alkali hydroxide to the fresh concrete [25,26,27,28,29]. Elevated temperatures were also adopted in these two methods to further accelerate the ASR deterioration. Immersion in solution is a practical approach only for small specimens in a laboratory environment.

Sanchez et al. [30] studied the ASR damage in concrete on 20 concrete mixtures with 10 different reactive aggregates. The target strengths of the concretes were 25 MPa, 35 MPa and 45 MPa. Concrete cylinders 100 × 200 mm were cured in 100% RH and 38 °C for compression testing. From each concrete, at least 35 cylinders were tested at different expansion levels. The test results of the cylinders from the twenty concrete mixtures revealed that the degradation of compressive strength is less than the other tested concrete properties, such as tensile strength and Young’s modulus. At 0.3% expansion, the maximum reduction of compressive strength reached 30%, while the reduction in tensile strength and elastic modulus were about 70% and 65%, respectively [30]. Esposito et al. [31] investigated the correlation between the concrete expansion due to ASR and the degradation of concrete mechanical properties. One type of reactive aggregate from Norway and one type of nonreactive aggregate from the Netherlands were used in two similar concrete mixtures. Concrete samples were cured at 96% RH and 38 °C. Expansion measurements were taken on 75 × 75 × 280 mm prisms. Compressive and split tensile strengths were determined using 150 mm cubes. The 100 × 100 × 400 mm prisms were tested for the elastic modulus. Among these properties, modulus of elasticity, with up to 90% degradation, was the best indicator of the ASR degradation. Despite some compressive strength gain below 0.3% expansion, the compressive strength, as well as the split tensile strength, decreased to about 60% of their undamaged values at expansions around 2.5%. Most of the split tensile strength reduction happened at an expansion below 0.6% [31]. Berra et al. [32] modeled the long-term ASR expansion of concrete structures. The alkali release from the aggregates was used in their model as obtained from a laboratory-accelerated alkali extraction test. The aggregate samples were subjected to leaching in an autoclave at 105 °C using a saturated calcium hydroxide solution. Accordingly, the developed model was used to predict the long-term ASR-induced expansion of a typical dam concrete mix and the results were consistent with the field measurements [32].

Another very important aging mechanism that may impact the performance of RC structures is the corrosion of rebar. The initiation of corrosion needs an anode for oxidation reactions, a cathode for the reduction of oxygen, an electrical connector to carry the electron charge and an electrolyte to carry the ionic charge [33]. When a chloride ion reaches the rebar, the stable passivized surface film acts as a cathode, and the decomposition of the passive layer starts. After the oxidation of the steel, hydroxide forms, which leads to corrosion where ferrous hydroxide becomes ferric hydroxide, followed by hydrated ferric oxide (rust). In RC, corrosion in rebar not only causes reduction in the steel cross-sectional area but also leads to the cracking and spalling of the concrete due to the growth of the oxide and rust [17,34]. In certain cases, it may even cause the brittle failure of the reinforcement [35], reduced bond strength [36] and a reduction in the reliability of the adjacent concrete [37].

Three methods or their combinations have commonly been adopted in literature to accelerate corrosion in RC: creating a corrosion cell and driving the corrosion by passing an electric current through rebar, submersion of specimens in chloride solutions with wet–dry cycles and the addition of chloride directly into the concrete in its fresh state. To create the corrosion cell, a wet sponge [35,36,37] or direct immersion into a saline solution has been adopted [38,39]. Passing a current is an effective approach for accelerating corrosion; however, it comes with various challenges for full-size structures because of the difficulties in creating the electrochemical cell and achieving a uniformly distributed corrosion in the reinforcement throughout the structure. Adding salts directly to fresh concrete showed good results in accelerating corrosion [38]. Pruckner and Gjørv [39] showed that adding sodium chloride (NaCl) to the concrete as a source of chloride results in a higher pH and lower Cl^−^/OH^−^ ratio compared to calcium chloride (CaCl_2_) addition. This has been verified by other investigations with a conclusion that the corrosive effect of NaCl is less than that of CaCl_2_ [39,40]. However, the impacts of directly adding chlorides into the fresh concrete on various properties of the resulting hardened concrete have not been studied in detail before.

Otieno et al. [41] performed a 2 ¼ year-long experiment, in which they exposed 105 beams, 120 × 130 × 375 mm in dimension, to accelerated laboratory corrosion under cycles of 3 days of wetting with 5% NaCl solution followed by 4 days air drying. During the same period, another 105 beams of the same dimensions were exposed to natural corrosion in a marine tidal zone. The test variables to be measured included the crack width induced by third point loading and the effect of concrete cover. The study could not establish a relationship between the accelerated and natural corrosion tests. For example, no general trend of the influence of the concrete crack width on the corrosion was found between the laboratory and field samples. Moreover, the effect of more concrete cover on the corrosion rate was less in the field samples compared to the laboratory ones [41]. Choe et al. [42] estimated the corrosion expansion rate of reinforcements in RC specimens through accelerated corrosion tests. A finite element analysis on the corrosion-induced concrete cracks under the influence of different confinement pressures from the transverse reinforcement was performed. It was found that a 2.0% loss of cross section corresponds to the critical corrosion of the rebar. Beyond this limit, the resulting concrete surface cracks required repair to prevent salt diffusion through the connected cracks [42]. Castaldo et al. [43] took a probabilistic computational approach to evaluate the service life of RC elements. The changes in mechanical and geometrical properties over time due to corrosion induced by chloride diffusion were predicted. The results are proposed to be used to evaluate the service lives of RC structures and to determine the optimal inspection and maintenance times during the life of the structure.

Both ASR and corrosion in RC structures manifests itself in the form of cracks, which have an adverse effect on the mechanical performance of the structure. Existing studies focused on mixture designs, sample size and curing and exposure conditions, among others, to understand the influence of these parameters on the ASR or corrosion rate. A major contribution of our study is in the understanding of the only practical accelerated aging method for large-scale studies (that is the direct addition of chemicals into the fresh concrete) on the properties of the ensuing concrete. This has been lacking in existing studies. Additionally, the influence of the batch size was also studied because it is important for large-scale studies. The results presented in this paper add to the existing datasets to improve the understanding of not only the ASR and corrosion degradation of concrete structures but also how accelerated aging studies can be conducted with minimal influence on the concrete properties to represent the field conditions.

The solution immersion method used in the literature consists of conditioning, fluid ingress and salt ion diffusion [44,45,46,47,48,49,50]. It has been proven that this method is time-consuming even for small concrete cylinders [51]. In this study, the effects of the direct addition of sodium hydroxide (total alkali loading varying from 2.98 kg/m^3^ to 5.57 kg/m^3^), for accelerating ASR and calcium chloride (from 1% to 4% by weight of cement) for accelerating corrosion, on the material properties of concrete were investigated. The measurements were performed on three series of specimens with different batching volumes (100 L, 2000 L and 4000 L). Expansion, cracking, bulk diffusivity, surface resistivity and compressive, split tensile and flexural strength tests were carried out to assess the efficiency of these two accelerated aging methods. The objective of this study is to expand the knowledge base regarding the acceleration of ASR and corrosion by the direct addition of alkaline and salt, respectively, into the fresh concrete as a practical method for the long-term performance evaluation of RC structures.

## 2. Experimental Program

### 2.1. Materials

**Figure 1:**
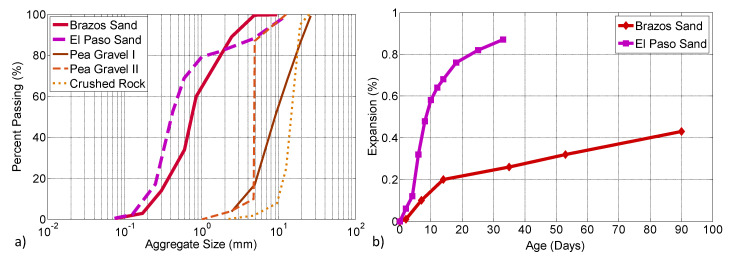
(**a**) Aggregate gradation and (**b**) expansion of Brazos River sand and El Paso sand based on ASTM C1260 (2014).

Both Type I/II and Type II cement were used in the concretes prepared for this study. Additionally, to suppress ASR in the specimens intended for accelerated corrosion study, Class F fly ash was used. The chemical compositions of cement and fly ash for these mixtures are given in Table 1. In order to deliberately accelerate ASR for laboratory trials, two types of reactive fine aggregates (from Brazos River, Houston, TX and El Paso, TX) were used. The coarse aggregates, pea gravel I, pea gravel II and crushed rock, with 19 mm, 9.5 mm and 19 mm nominal sizes, respectively, were regular and non-reactive. The gradations of coarse and fine aggregates are shown in Figure 1a. The size of coarse aggregate ranged from 1 mm to 20 mm, and the reactive fine aggregate showed a particle size mainly distributed between 0.1 mm and 10 mm. The potential alkali reactivity of the fine aggregate was evaluated via a mortar-bar test according to ASTM C1260 [52]. As shown in Figure 1b, the expansion of the Brazos sand exceeded 0.2% at 14 days, suggesting that the fine aggregate used in this work was reactive. Due to the high reactivity, additional mortar expansion due to ASR with a linear uptrend was observed and an expansion higher than 0.4% was obtained after 90 days. The El Paso sand showed a higher reactivity of above 0.6% and 0.8% expansion at 16 and 25 days of age, respectively. NaOH and CaCl_2_, with respective purities of 96% and 85%, were used as alkali and chloride resources to accelerate ASR and rebar corrosion, respectively. Analytical grade calcium hydroxide (Ca(OH)_2_) and NaCl with a purity of 99% were employed to make saturated lime water and a chloride solution for the bulk diffusivity test.

### 2.2. Mixture Proportions and Specimen Preparation

The proportions of the ten concrete mixtures prepared in this study are provided in Table 2. To study accelerated ASR in concrete, four concrete mixtures were prepared with Type I/II cement and reactive fine aggregate from Brazos River, and two concrete mixtures were prepared with Type II cement and reactive fine aggregate from El Paso. Different levels of NaOH addition were used in four concrete mixtures from Type I/II cement and Brazos River reactive sand. One of the mixtures was a regular self-consolidating concrete (SCC) with no fly ash and additional alkali (NaOH-0). Another three mixtures (NaOH-2, NaOH-4 and NaOH-8) had additional alkali hydroxide in the form of NaOH powder, which was directly added to the fresh concrete to raise the total alkali loading to 2.98 kg/m^3^, 3.84 kg/m^3^ and 5.57 kg/m^3^, which was calculated according to

(1) $$Total\;alkali\;content=f_{c}\;\left(\frac{kg}{m^{3}}\right)\times \left[Na_{2}O_{e}\;\left(wt.\%\right)+A_{a}\;\left(wt.\%\right)\right]$$

where *f*_c_ is the fraction of cement in the concrete, Na_2_O_e_ is the alkali content of the cement and *A*_a_ is the added alkali. Two concrete mixtures were made with Type II cement and El Paso reactive sand, from which the total alkalinity of one of the mixtures (NaOH-12) was increased to 5.3 kg/m^3^ using NaOH to accelerate ASR. The other concrete mixture (NaOH-00) did not contain additional alkali and its only source of alkali was the alkali content of Type II cement, as presented in Table 1. Corrosion was accelerated by adding 1%, 2% and 4% of chloride ions (in the form of CaCl_2_ flakes) by weight of cement to the fresh SCC mixture (with fly ash). CaCl_2_ was preferred over other salts because it induces higher corrosion rates, as discussed in Section 1. A control mixture with no chloride addition (labeled as SCC) was also prepared.

The concretes with a total alkali loading of 1.7 kg/m^3^ from Type II cement (0% NaOH addition by weight of cement), 2.1 kg/m^3^ from Type I/II cement (0% NaOH addition by weight of cement), 2.98 kg/m^3^ (0.2% NaOH addition by weight of cement), 3.84 kg/m^3^ (0.4% NaOH addition by weight of cement), 5.57 kg/m^3^ (0.8% NaOH addition by weight of cement) and 5.3 kg/m^3^ (1.25% NaOH addition by weight of cement) are referred to as NaOH-00, NaOH-0, NaOH-2, NaOH-4, NaOH-8 and NaOH-12, respectively, in the remainder of this paper. Similarly, mixtures containing 0%, 1%, 2% and 4% chloride ion by weight of cement are referred to as SCC, CaCl_2_-1, CaCl_2_-2 and CaCl_2_-4, respectively. Specimens were prepared in 13 separate casts. In addition to the eight casts for the eight mixtures from Type I/II cement and Brazos River sand shown in Table 2, SCC, NaOH-8 and CaCl_2_-4 mixtures were repeated using a larger batch size to study the effect of mixing volume on the material properties. The batching volume for the larger series (only SCC, NaOH-8 and CaCl_2_-4) was 2000 L, while the same for the smaller series was 100 L. The remaining two casts (one from each of the NaOH-00 and NaOH-12 mixtures) were made using Type II cement and the proportions shown in Table 2. The batching volume for the NaOH-00 and NaOH-12 series was 4000 L each.

The eight mixtures from Type I/II cement and Brazos River reactive sand had water-to-binder and fine-to-coarse aggregate ratios of 0.5 and 0.89, respectively. One type of coarse aggregate, pea gravel, with a nominal size of 19 mm, was used in these mixtures. A water-reducing admixture (Pozzolith 200N) and a high range water-reducing superplasticizer (Glenium 7500N) were used. The two mixtures from Type II cement and El Paso reactive sand had water-to-binder and fine-to-coarse aggregate ratios of 0.53 and 0.58, respectively. Two types of coarse aggregates, pea gravel and crushed rock, with nominal sizes of 9.5 mm and 19 mm, were used in these two mixtures. A water-reducing admixture (Pozzolith 8) and a high range water-reducing superplasticizer (Glenium 3400 NV) were used.

**Figure 2:**
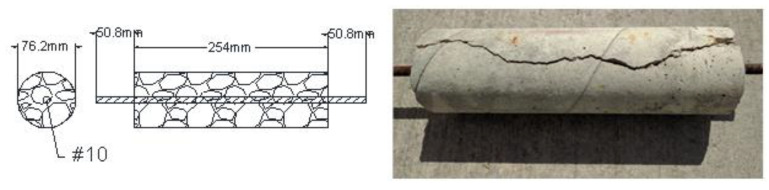
Cylindrical specimens with a single rebar for corrosion and cracking studies.

From the mixtures with Type I/II cement and Brazos sand, sixteen 75 × 150 mm cylinders were cast for the smaller batch for crack, density and absorption measurements. In total, four hundred and forty-two 100 × 200 mm cylinders for compression and split tension tests were cast. Four 100 × 200 mm cylinders were cast from the smaller batch for chloride diffusion tests. Forty-five 100 × 200 mm cylinders, with 13 cylinders from the smaller batch and 32 from the larger batch, were cast for surface resistance and corrosion potential tests. As shown in Figure 2, a series of concrete cylinders and cylindrical specimens with a single embedded soft Metric #10 (U.S. #3) rebar of 9.5 mm diameter were prepared. The diameter of the concrete cylinder was chosen based on the commonly used cover thickness in RC structures. The specimens were cast and stored outside under ambient environmental conditions in Houston, Texas. During the exposure, the temperature varied between 4 °C and 37 °C, and the RH varied between 30% and 100%. From the mixtures with Type II cement and El Paso sand, one hundred and eighty 100 × 200 mm cylinders were cast for compressive and tensile strength tests, forty-two 150 × 150 × 525 prisms were made for flexural strength tests and twenty prisms were cast for expansion measurements. The specimens experienced three different environments. A group of specimens were kept in the lab environment. A group of specimens were kept under a tarp to maintain moisture and water was sprayed twice a week while in the outside environment of Los Angeles, California. Continuous measurements were taken, indicating that the temperature and RH of these specimens were respectively maintained between 9 °C to 37 °C and 80% to 100% over the exposure period. A group of specimens were stored in an environmental chamber at 50 °C and 90% RH.

### 2.3. Characterization Methods

The testing plan and standards for specimens from concretes with Type I/II cement and Brazos River reactive sand in larger mixing volume size (i.e., 2000 L) and smaller mixing volume size (i.e., 100 L) are summarized in Table 3 and Table 4, respectively. At least three specimens from each set were tested at each age. The testing plan and standards for specimens from concretes with Type II cement and El Paso reactive sand are summarized in Table 5. At each age, three specimens were tested for compressive and tensile strength and two specimens were tested for flexural strength.

Density, void and absorption were measured at 28 days following ASTM C642 [61], which involved the gravimetric density measurement of 75 × 100 mm cylinders after different processes such as oven drying, water saturating and boiling in water. Crack formation and propagation were monitored based on visual examination. The surface of the specimens was divided into multiple similar sections and the development of cracks in each section was recorded to obtain the crack maps. The crack width was measured using a magnifier with 0.1 mm scale. To have a better understanding of the width of the cracks less than 0.1 mm, a picture was taken using a high-resolution digital camera and graduated magnifier. The digital photo of the crack was imported into a computer-aided design (CAD) software and, based on the scale in the picture, the crack width was calculated.

**Figure 3:**
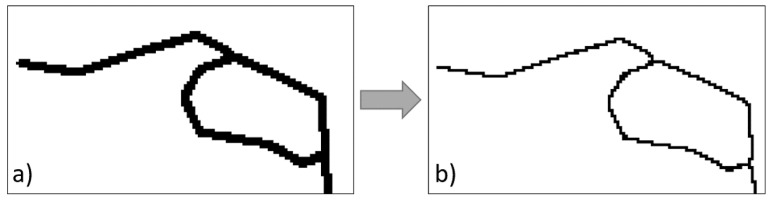
Preprocessing of images: (**a**) binarization and (**b**) shrinkage.

An image processing method, as shown in Figure 3, was used to monitor the development of the cracks on the surface of the cylinders. The crack patterns were recorded on the surface of the cylinders at specific time intervals. To quantify the intensity of the cracks, a density parameter was proposed, which is the sum of the crack length divided by the measurement area. Furthermore, the length of the crack and the measurement area were calculated in terms of pixels counts; thus, no conversion of the units was necessary. The crack density was obtained in units of 1/pixels. A computer code using MATLAB [63] was developed to calculate the crack density where the number of the black pixels were counted and divided by the total number of pixels in the image. Preprocessing steps, including contrast enhancement and binarization were performed to minimize the effect of image quality on the result. In addition, a morphological transformation was conducted to shrink the cracks. After this operation, the cracks with different variable widths had the same unit width and therefore only the crack growth in terms of the length could be calculated (see Figure 3).

The surface resistivity test was performed on 100 × 200 mm concrete cylinders according to AASHTO TP 95-14 [59]. The specimens were water saturated for 7 days prior to testing. Four four-probe arrays on the surface of the specimen at 90° from each other were used for the resistivity measurement. The surface resistivity is related to chloride penetration and a higher surface electrical resistivity indicates a higher chloride penetration resistance. According to AASHTO TP 95-14 [59], concretes with the following ranges of surface resistivity: less than 12 kΩ-cm, 12–21 kΩ-cm, 21–37 kΩ-cm, 37–254 kΩ-cm and more than 254 kΩ-cm are respectively classified as having high, moderate, low, very low and negligible chloride penetration. The surface resistivity was measured on a monthly basis.

A bulk diffusivity test for ASR specimens was performed based on NT-Build 443 [57]. After 28 days, 100 × 200 mm concrete cylinders were cut in half and immersed in a saturated calcium hydroxide solution until their weights have stabilized (no more than 0.1% change within a 24-h period). All the surfaces, except the one to be exposed, were coated with epoxy to create a 1-D diffusion case. Then, these specimens were divided into three groups and stored in a NaCl solution (165 ± 1 g NaCl per dm^3^ of water) for 28 days, 180 days and 365 days. The specimens were then ground in layers and a small amount of concrete powder was collected from different depths. The amount of chloride in these concrete powder specimens was measured using titration according to NT-Build 208 [56]. The amount of chlorides (Cl^−^) was calculated according to

(2) $$\%{\mathrm{Cl}}^{-}=3.545\frac{V_{1}N_{1}-V_{2}N_{2}}{m}$$

where *V*_1_ is the added amount of silver nitrate solution (mL), *N*_1_ is the normality of the silver nitrate solution, *V*_2_ is the amount of ammonium thiocyanate solution added during the titration (mL), *N*_2_ is the normality of the ammonium thiocyanate solution and *m* is the weight of the specimen (g).

To calculate the effective chloride transport coefficient, a general form of Fick’s second law with the assumption of constant external chloride concentration was used

(3) $$C\left(x,t\right)=C_{s}-\left(C_{s}-C_{i}\right)∙\mathrm{erf}\left(\frac{x}{\sqrt{4D_{e}t}}\right)$$

where *C(x,t)* is the chloride concentration as a percentage of grams of chloride per gram of concrete (wt.%), *C*_s_ is the surface chloride concentration (wt.%), *C*_i_ is the initial chloride concentration measured in the concrete (wt.%), *x* is the depth below the exposed surface (m), *D*_e_ is the effective chloride transport coefficient (m^2^/s), *t* is the exposure time (s) and *erf* is the error function.

In order to determine the corrosion activity of the embedded steel rebar, open circuit corrosion potential measurements were performed monthly from 28 days on the SCC and CaCl_2_-X cylindrical specimens (where X represents the different percentages of CaCl_2_ addition) with an embedded #10 rebar according to ASTM C876 [58]. In RC, concrete acts as an electrolyte and the reinforcing steel bar develops a potential depending on the concrete environment [64]. The principle involved in this technique is essentially the measurement of the corrosion potential of the reinforcing steel bar based upon the half-cell reaction, Cu → Cu^2+^ + 2e^−^, corresponding to the potential of the saturated copper/copper sulfate reference electrode (CSE), with the reference to the hydrogen electrode being −0.30 V at 22.2 °C. The copper/copper sulfate reference electrode has a temperature coefficient approximately 0.0005 V more negative per Fahrenheit degree for the temperature range from 0 °C to 49 °C [65]. In the testing process, the solution in the reference electrode makes simultaneous electrical contact with the porous plug and the copper rod at all times. A tablet/smartphone-based nondestructive testing device with a voltage measurement range from −1000 mV to 1000 mV and an accuracy of 0.1 mV was used. In this study, since carbonation was not observed on the specimens, the temperature and humidity of the specimens were recorded alongside the potential. To perform the measurement, initially the surface of the exposed rebar was brushed to ensure a low electrical resistance connection from a bright metal to a bright metal. Afterwards, the test clip was directly connected to the reinforcing bar. In the next step, based on the initial measured value, the surface of the concrete was prepared with pre-wetting. The potentials on the surface of the concrete, with respect to an arbitrary reference point on the reinforcement, was measured by placing the probe on the surface of the concrete at different locations. According to ASTM C876 [58], the probability of corrosion is less than 10%, uncertain and more than 90% for potential values higher than −200 mV, potential values in the range from −200 mV to −350 mV and potential values lower than −350 mV, respectively. Similar to surface resistivity tests, the corrosion potential was measured on a monthly basis.

The compressive strength and split tensile strength tests were carried out on cylindrical specimens of 100 × 200 mm according to ASTM C39 [53] and C496 (2017), respectively. At least three repetitions were employed for each test. A stress rate of 0.25 ± 0.05 MPa/s was adopted in the compression test. Both the stress and strain of the concrete (using an axial averaging extensometer) were recorded. A loading rate of 1.0 MPa/min was used in the split tension tests. The splitting tensile strength, *S*_T_, of the specimen was calculated according to:

(4) $$S_{T}=\frac{2P}{\pi lD}$$

where *P* is the maximum load indicated by the testing machine, *l* is length and *D* is diameter the specimen.

The flexural strength tests were carried out on prism specimens of 150 × 150 × 525 mm dimensions according to ASTM C78 [62]. There were two repetitions for each age and material. A constant stress rate of 1.05 MPa/min was applied on the tension face of the prisms. The modulus of rupture, *R*, of the prism was calculated according to:

(5) $$R=\frac{PL}{bd^{2}}$$

where *P* is the maximum applied load indicated by the testing machine, *L* is the span length, *b* is the average width of the specimen and *d* is the average depth of the specimen.

**Figure 4:**
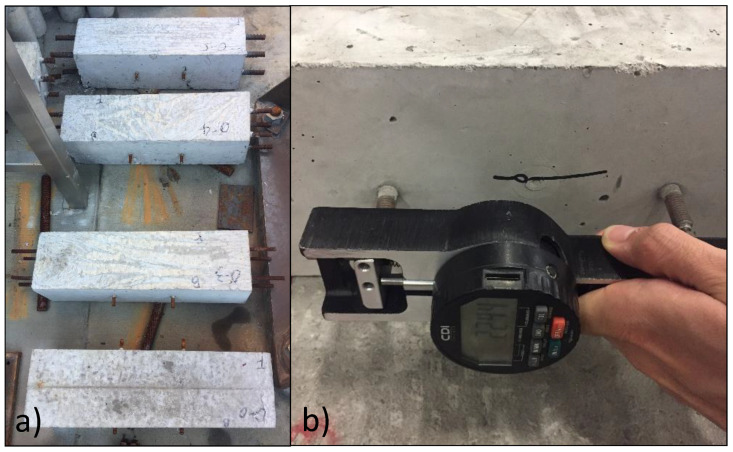
Expansion measurements on the prisms from NaOH-00 and NaOH-12 mixtures: (**a**) one group of prisms and (**b**) demountable mechanical (DEMEC) strain gauge device.

Expansion measurements were performed on 150 × 150 × 525 mm prisms made from NaOH-00 and NaOH-12 mixtures. Prisms were reinforced with longitudinal reinforcement ratios of 0% with no rebar and 1.23%, 2.18% and 3.41% using different sizes of rebars having 9.525 mm, 12.7 mm and 15.875 mm diameter, respectively, close to the four corners of the section, as shown in Figure 4a. A demountable mechanical (DEMEC) strain gauge, with a 15.25 mm gauge length and a 0.002 mm resolution, was used to take the expansion measurements, as shown in Figure 4b.

## 3. Results and Discussion

### 3.1. Density, Void and Absorption

The results of the measurements at 28 days are shown in Table 6. In the presence of sodium hydroxide, the change in the density of the concrete was less than 4.9%. The absorption and voids of the concrete decreased first and then increased with increasing amounts of NaOH. At a total alkali loading of 2.98 kg/m^3^ and 3.84 kg/m^3^, the absorption decreased by 3% and 9%, and the voids decreased by 5% and 9%, respectively. Meanwhile, the alkali loading of 5.57 kg/m^3^ resulted in similar results to the control specimens. The decrease in the absorption and voids in NaOH-2 and NaOH-4 is attributed to the modification of cement hydration and the corresponding hydration products. As the amount of alkali increased, a less dense microstructure is obtained due to the formation of a more reticular and porous micro-texture. The chloride addition resulted in a negligible variation in the density of concrete of about 4.9%.

### 3.2. Cracking Observations

**Figure 5:**
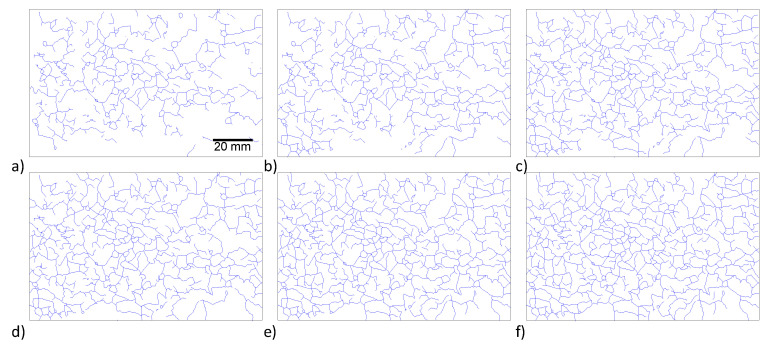
Alkali-silica reactivity (ASR) crack propagation on a 75 × 150 mm cylinder of the larger batch series NaOH-8 mixture (**a**) 114 days, (**b**) 171 days, (**c**) 225 days, (**d**) 342 days, (**e**) 342 days and (**f**) 432 days after casting.

**Figure 6:**
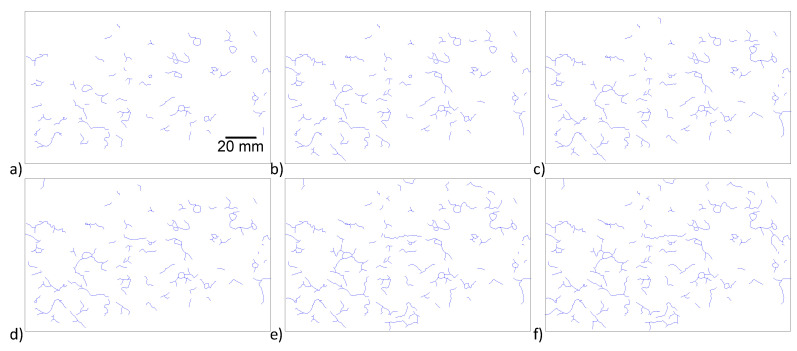
Alkali-silica reactivity (ASR) crack propagation on a 100 × 200 mm cylinder of the larger batch series NaOH-8 mixture (**a**) 114 days, (**b**) 171 days, (**c**) 225 days, (**d**) 342 days, (**e**) 342 days and (**f**) 432 days after casting.

In the specimens with accelerated ASR, the expansion of the ASR gel around the aggregate resulted in micro-cracks which, over time, propagated and appeared on the surface of the specimens. For the larger batch series of NaOH-8 specimens, the first map cracks were observed after approximately 90 days. Over the course of time, the density of cracks increased and the cracks started to coalesce. Figure 5 and Figure 6 show the crack maps at six different ages for 75 × 150 mm and 100 × 200 mm specimens, respectively, from the larger batch series with a total alkali loading of 5.57 kg/m^3^. A comparison of the cracks on the specimens with two different sizes indicated that the geometry plays an important role in the amount of cracking, and smaller specimens showed more cracks on the surface. These results highlight the important effect of specimen geometry on the accelerated aging testing. The crack widths were measured, and it was observed that they remained almost constant (from 0.05 mm to 0.1 mm) during the testing period.

**Figure 7:**
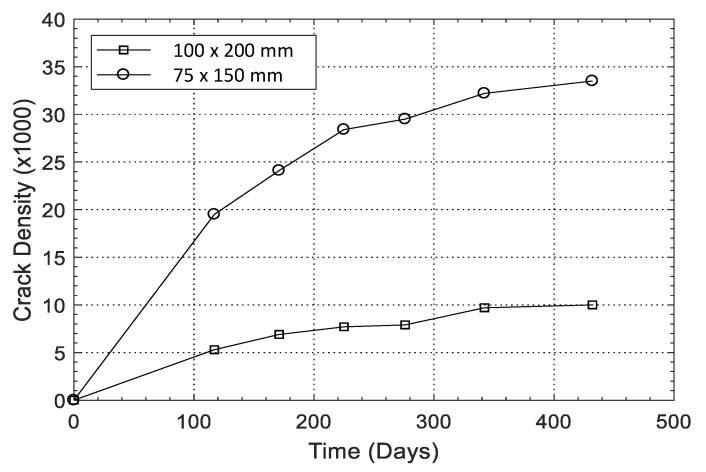
Crack density versus time for NaOH concrete cylinder samples.

Size effect was observed in the results, which means that the crack density is a function of the specimen size. Both samples were of the same concrete mixture and were maintained in the same environmental conditions. In the smaller cylinders, the cracks were fully joined together, and further expansion did not create additional cracks, unlike the larger cylinders. As shown in Figure 7, after 117 days, from the smaller specimens (75 × 150 mm), an average crack density of 19.5 × 10^−3^ was observed and this value kept increasing over time and reached to 33.5 ×10^−3^ at 432 days. The larger specimen showed a reduced crack density. At the same testing times (117 days and 432 days), the average crack density of the 100 × 200 mm cylinders was 73% and 70% less than that of the small specimens.

**Figure 8:**
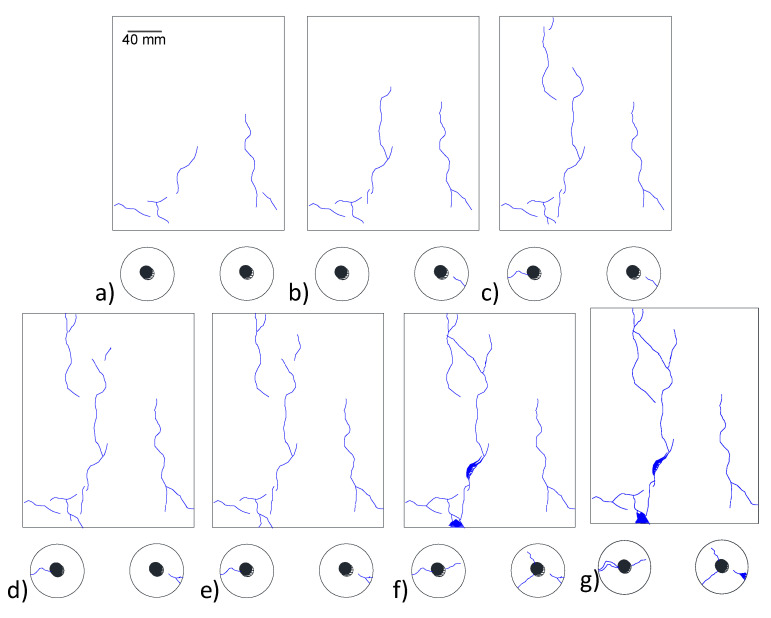
Crack formation due to corrosion expansion on a CaCl_2_-4 specimen of the larger batch series with a soft Metric #10 rebar at (**a**) 117 days, (**b**) 171 days, (**c**) 225 days, (**d**) 276 days, (**e**) 342 days, (**f**) 432 days and (**g**) 492 days after casting.

**Figure 9:**
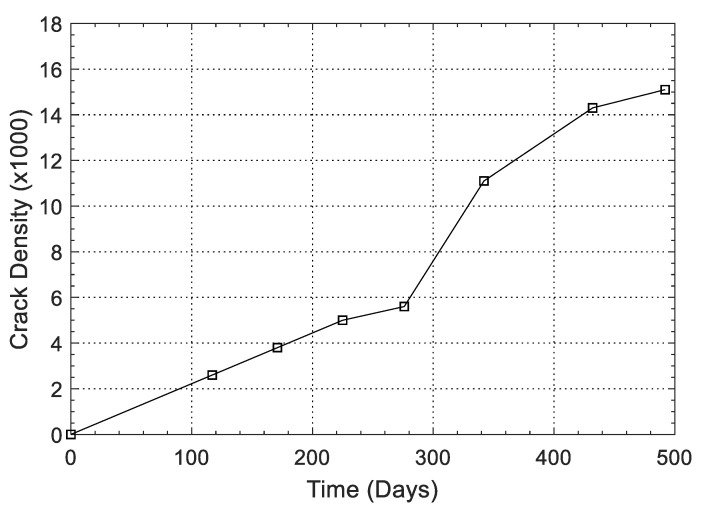
Crack density versus time for the CaCl_2_-4 specimen of the larger batch series.

Results from the visual inspection of cylindrical specimens made of CaCl_2_-4 concrete of the larger batch series with an embedded soft Metric #10 rebar are shown in Figure 8. Cracks started to appear on the specimen surface approximately 90 days after casting. The crack widths were continuously measured on two cylindrical specimens and the maximum crack width was observed to grow from 0.6 mm at 117 days to 4.2 mm at 492 days. As seen in Figure 8, spalling at certain locations was observed. Mass loss of rebar in two specimens was measured according to ASTM G1 [55]. At 28 days, a 1.27% mass loss was observed, which increased to 2.02% at 365 days. Figure 9 shows the development of crack density as a function of time. It can be seen that the crack density in the first 276 days increased slowly with a value lower than 6 × 10^−3^. However, the crack density experienced a rapid increase after 276 days and this value at 592 days increased to 15.1 × 10^−3^. The total pixel density was not calculated; however, as shown in Figure 5, Figure 6 and Figure 8, a line that represents a specific length, i.e., 20 mm or 40 mm, was detected and the crack width was calculated during the measurement time. These results demonstrate that, by adding 4% calcium chloride, concrete damage in the form of cracking starts as early as 100 days and continues unless a mitigation strategy is adopted. To assess the influence of chloride addition on the corrosion activity of the embedded steel rebar, open circuit corrosion potential measurements were performed, as shown in Section 3.4.

### 3.3. Effect of ASR on Diffusivity

**Figure 10:**
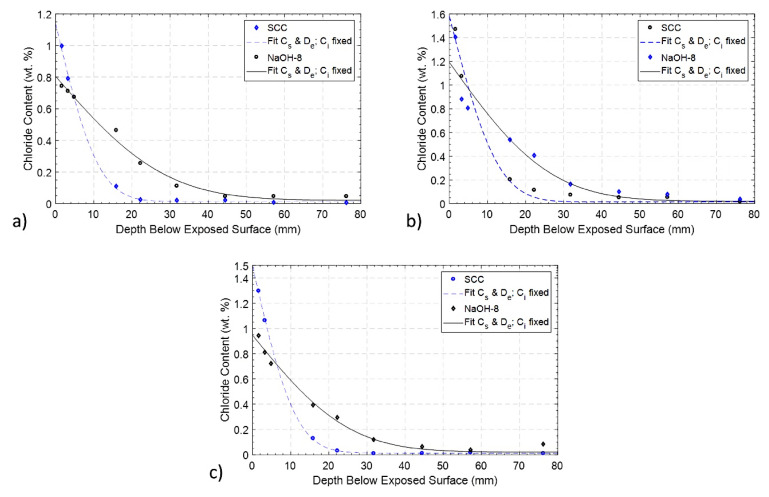
Chloride diffusion characterization of the larger batch series of SCC and NaOH-8 concrete specimens at (**a**) 28 days, (**b**) 210 days and (**c**) 365 days after casting.

The bulk diffusivity test was performed on the larger batch series of SCC and NaOH-8 mixtures only. The results of these tests are shown in Figure 10. It is seen that for the concrete with fly ash and no NaOH addition (i.e., the SCC mixture), the diffusivity is lower than the mix without fly ash and a total alkali loading of 5.57 kg/m^3^ (0.8% NaOH addition). This difference is attributed to the presence of NaOH but more importantly to the reduced diffusivity of the SCC mixture due to the presence of fly ash [66,67,68]. As shown in Table 7, the diffusivity of the SCC mixture increased by 22.4% and 14.1% after 210 days and 365 days, respectively, in comparison with its value at 28 days. The slight decrease from 210 days to 365 days is due to the difference in the amount of chloride on the concrete surface of different specimens, which is difficult to measure in the layered grinding processes after ponding, and has a great influence on the ultimate diffusivity values. The diffusivity of the NaOH-8 mixture increased by 50% at 365 days in comparison to the measurement at 28 days. This increase is related to the increased interconnectivity of micro-cracks inside the concrete. ASR results in microcracking, voids and a detachment at the cement paste–aggregate boundary, increasing the overall porosity of the mortar [69]. These defects inside the concrete provide additional and direct pathways for chloride ingress and diffusion, leading to a higher chloride content at larger depths.

### 3.4. Effect of Chloride on Corrosion Activity of Steel Rebar

**Figure 11:**
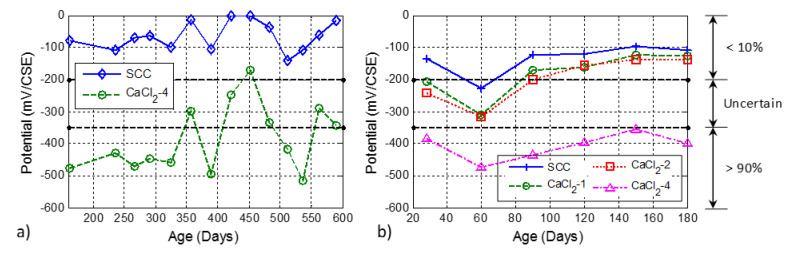
Open circuit corrosion potential of (**a**) SCC and CaCl_2_-4 mixtures from the larger batch series and (**b**) CaCl_2_ mixtures from the smaller batch series. Note that the horizontal dashed lines are the limits in ASTM C876 (2015).

The development of open circuit corrosion potential as a function of time for the larger batch series of CaCl_2_-4 and SCC mixtures is shown in Figure 11a. The corrosion limits as per ASTM C876 [58] are also shown in Figure 11 with horizontal dashed lines. It is seen that without any addition of chloride ion, the open circuit corrosion potential value of SCC was always higher than −200 mV, which indicates that the probability of corrosion is low (<10%). Contrary to SCC, the specimen with 4% additional chloride ions exhibited a high probability of corrosion (>90%) for most of the testing time. Higher deviations in the measurement are observed for the larger batch specimens compared to the smaller batch series, which might be caused by the high sensitivity of the probe to the surface moisture. It is noted here that all the specimens were kept outside in the environmental conditions of Houston, Texas which varies substantially in terms of RH and temperature. The specimens from the smaller batch series showed a similar trend with a lower amount of variation. It is seen in Figure 11b that the probability of steel corrosion increased with the increasing amount of chloride. It was also found that the effect of adding 1% and 2% chloride ion by weight of cement to the mixture was almost the same. However, 4% chloride increased the probability of corrosion to more than 90%. The results obtained from the smaller batch series indicate that the corrosion of the embedded steel rebar occurs at with a very high probability at a chloride level of 4%, while the specimens with 1% and 2% chloride also showed the initiation of corrosion at certain test ages. It is noted that these observations were obtained in the ambient environmental conditions of Houston, Texas, and might not be applicable to indoor laboratory investigations or field tests with different conditions.

### 3.5. Surface Resistivity

**Figure 12:**
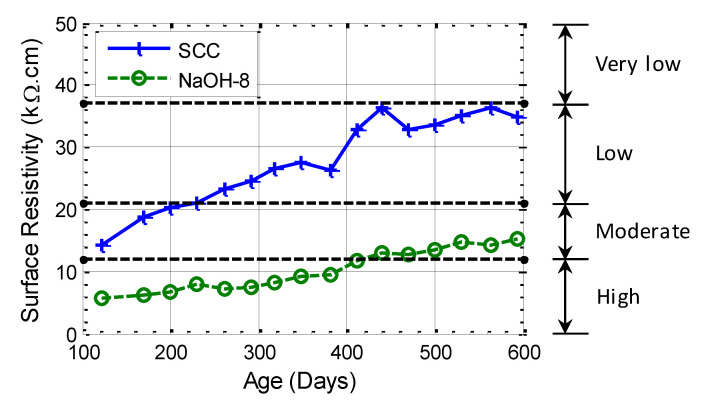
Effect of ASR on surface resistivity: NaOH-8 and SCC from the larger batch series. Note that the horizontal dashed lines are the limits in AASHTO TP 95-14 [59].

A comparison of the larger batch series of NaOH-8 and SCC mixtures in Figure 12 showed that SCC had a higher surface resistivity (or less diffusivity to chloride) and its difference from the NaOH-8 mixture grows over time. This observation indicates that the presence of fly ash suppresses ASR and, although the surface resistivity of the NaOH-8 mixture increased with time, the rate of increase was lower compared to the SCC mixture. This is attributed to the ASR damage in the NaOH-8 specimen that reduced the improvement due to hydration. Three of the AASHTO TP 95-14 [59] limits for surface resistivity are also shown in Figure 12 using horizontal dashed lines.

**Figure 13:**
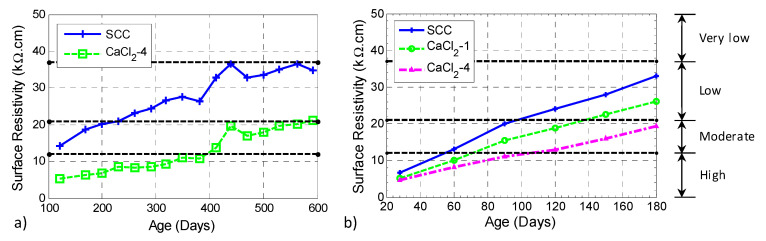
Effect of rebar corrosion on surface resistivity in (**a**) SCC and CaCl_2_-4 mixtures from the larger batch series and (**b**) CaCl_2_ mixtures from the smaller batch series. Note that the horizontal dashed lines are the limits in AASHTO TP 95-14 [59].

Chloride content is normally considered as one of the parameters that affects the surface resistivity. The presence of chloride in concrete decreases the surface resistivity because the electrons are carried through the negatively charged ions in the concrete pore solution in saturated conditions [70]. The results for the larger batch series of specimens are shown in Figure 13a. It is seen that the addition of chloride ion results in a reduction in surface resistivity. A similar trend can be observed from the results shown in Figure 13b: the surface resistivity of concrete decreases with increasing amount of chloride. The results of CaCl_2_-2 were not consistent with the rest of the data, which might have been caused by random concentrations of the chloride ion close to the surface. Therefore, this group is not presented here. It is seen that, without the addition of chloride salt, the chloride penetrability of SCC for the smaller batch of specimens was high in the first 50 days, moderate between 50 and 100 days, and then became low. However, by mixing 4% chloride ion by weight of cement, the chloride penetrability was high in the first 100 days and then became moderate. The surface resistivity results indicate that, after 180 days, the chloride penetrability of SCC, CaCl2-1 and CaCl2-4 was in the range of low, low and moderate, respectively.

### 3.6. Expansion Measurements

**Figure 14:**
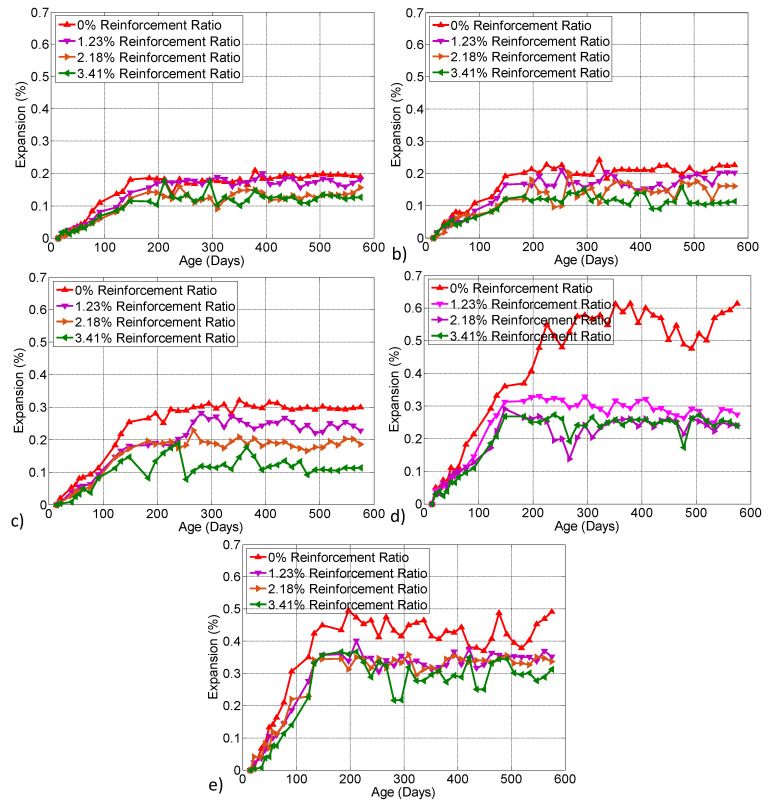
Expansion of prisms built from (**a**) NaOH-00 mixture and kept in the laboratory, (**b**) NaOH-00 mixture and conditioned outside, (**c**) NaOH-00 mixture and conditioned in the environmental chamber, (**d**) NaOH-12 mixture and conditioned outside and (**e**) NaOH-12 mixture and conditioned in the environmental chamber.

The results of the longitudinal expansion measurements on the prisms from NaOH-00 and NaOH-12 are presented in this section. Each data point is the average of two measurements taken from one prism. Figure 14a–e present the expansion results of the prisms, respectively, built from NaOH-00 kept in the laboratory, from NaOH-00 and conditioned outside, from NaOH-00 and conditioned in the environmental chamber, from NaOH-12 and conditioned outside and from NaOH-12 and conditioned in the environmental chamber. Each graph includes four datasets, one for each reinforcement ratio. The 0.24% final expansion of the prisms built from NaOH-00 and conditioned outside was found to be slightly more than the 0.21% expansion of the prisms built from NaOH-00 and kept in the laboratory. On the other hand, the 0.62% final expansion of the prisms from NaOH-12 with a 5.3 kg/m^3^ total alkali content and conditioned outside was about three times the average expansion of 0.22% of the prisms built from NaOH-00. High temperature and RH caused the expansions of the prisms in the environmental chamber to be higher than those in the lab or outside, with one exception. The exception occurred for the 0.49% final expansion of the plain concrete prism from the NaOH-12 mixture in the environmental chamber compared with the 0.62% expansion of similar prisms conditioned outside. This higher expansion of the prisms conditioned outside is attributed to the wet and dry cycles. The prisms from the NaOH-12 mixture conditioned in the environmental chamber showed a 0.49% final expansion while the other prisms in the chamber built from the NaOH-00 mixture showed a 0.34% expansion. The 0.34% expansion of the plain concrete prism from NaOH-00 shows that in an environment with high temperature and RH, even concretes with a low alkali content may experience a high level of ASR in the presence of reactive aggregates.

**Figure 15:**
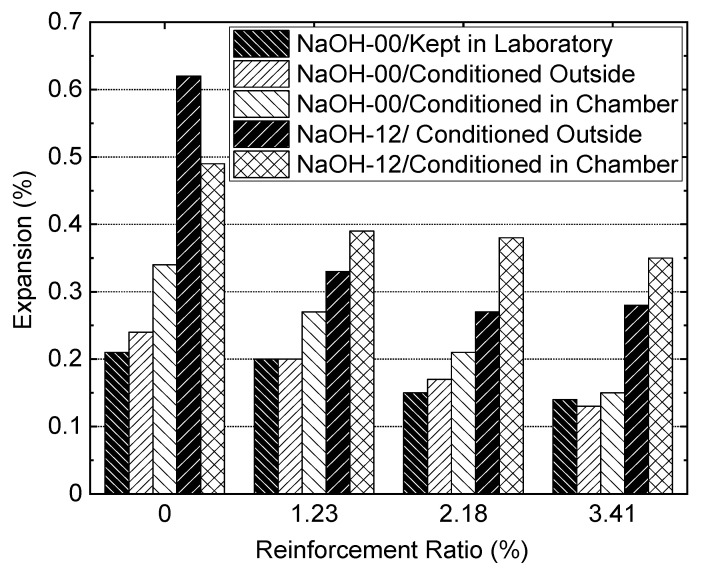
Expansion of prisms with different reinforcement ratios.

Logarithmic regression analysis was performed on the expansion data points shown in Figure 14. The R^2^ coefficient of the regression was found to vary between 0.8 and 0.9 for different datasets. The final expansions of prisms obtained from the regression at 575 days of age are summarized in Figure 15 for different reinforcement ratios. The expansion due to ASR was counteracted to some extent when internal reinforcement was added to the prisms. For prisms from NaOH-00 and NaOH-12 in different environments, the highest reinforcement ratio of 3.41% reduced the expansions by 29% to 56% compared to those from plain concrete. For the NaOH-00 mixture, the prisms with 1.23% and 2.18% reinforcement ratios kept in the laboratory reduced the expansion by approximately 5% and 29%, respectively, compared with the plain concrete prisms of the same mixture. In the outside environment for the NaOH-00 mixture, the prisms with 1.23% and 2.18% reinforcement ratios reduced the expansion by approximately 17% and 29%, respectively, compared with the plain concrete prisms. The 1.23% and 2.18% reinforcement ratios also reduced the final expansion of prisms from the NaOH-12 mixture conditioned outside to about half of the expansion of the plain concrete prisms from the same mixture. In the environmental chamber, the prisms from NaOH-00 with 1.23% and 2.18% reinforcement ratios, respectively, reduced the final expansion by about 21% and 38% compared with the expansions of the plain concrete prisms. This expansion reduction was about 20% for the prisms from NaOH-12 with 1.23% and 2.18% reinforcement ratios compared to the plain concrete prisms in the chamber.

### 3.7. Compressive Strength

**Figure 16:**
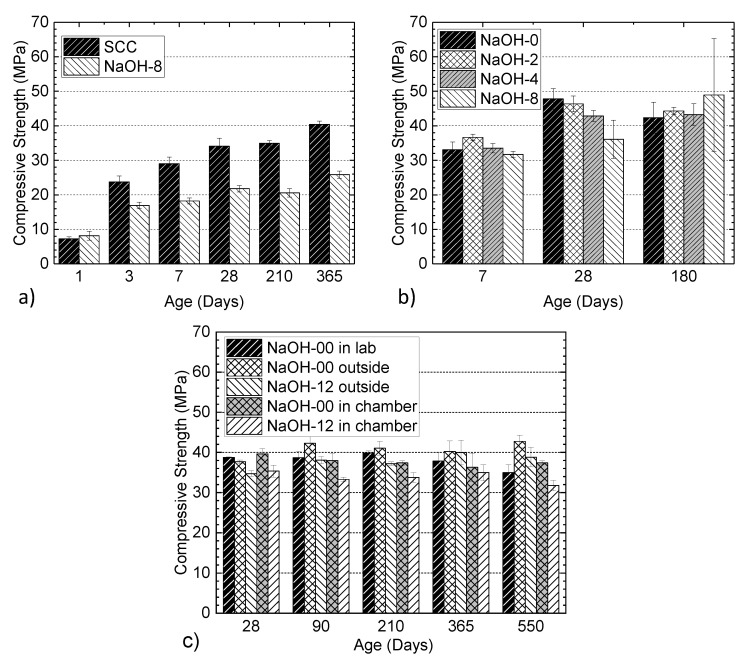
Effect of alkali addition on the compressive strength of concrete: (**a**) NaOH-8 and SCC mixtures from the larger batch series, (**b**) NaOH mixtures from the smaller batch series and (**c**) NaOH-00 and NaOH-12 mixtures.

**Figure 17:**
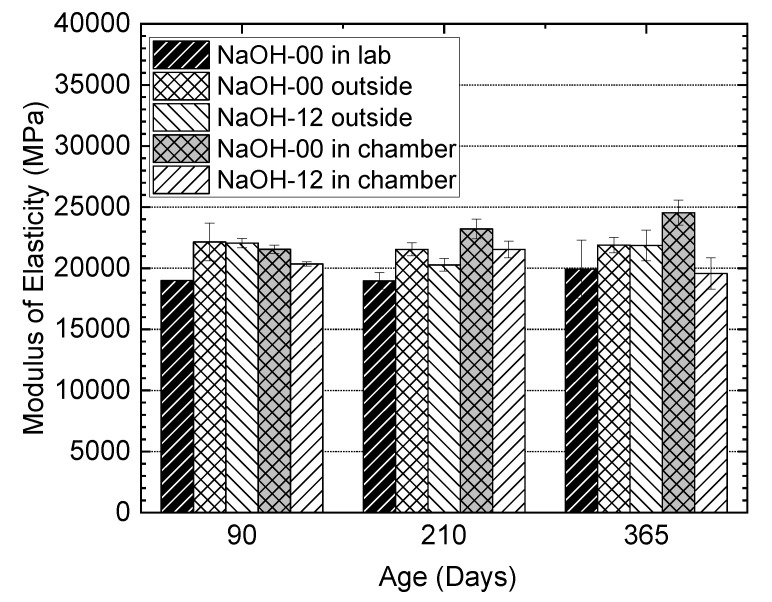
Effect of alkali addition on the elastic modulus of concrete at different ages.

Figure 16a shows a comparison of the compressive strengths of SCC and NaOH-8 mixtures from the larger batch series. Testing was performed over a longer period of time than the smaller batch series and the results indicated a lower compressive strength of NaOH-8 in comparison to the SCC mixture. These two mixtures exhibited a similar day one strength; however, a lower strength was shown by NaOH-8 for most of the experimental program period. This is attributed to a more reticular and porous cement paste in the presence of alkali [71] and the defects in concrete induced by the ASR expansion. The literature indicates that the effect of ASR mostly manifests itself in the modulus of the elasticity of concrete, while the impact on the strength is rather small [72]. As shown in Figure 16b, NaOH-2 had a slightly higher strength at day one due to an increase in the hydration reactions compared to the control specimens. However, further addition (NaOH-4 and NaOH-8) was not beneficial in this regard. NaOH-4 showed a very similar day seven strength to the control mixture while NaOH-8 had slightly lower compressive strength. In the presence of moderate amounts of alkali, the initial hydration of cement is enhanced; however, the reactions and strength development are retarded [73,74]. At 28 days, the control mixture with no NaOH had the highest strength and the strength gradually became lower at higher NaOH levels. The results for 180 days should be interpreted with the consideration of the variation in the measured values. This variation might also be attributed to the densified paste of concrete at the early stage of ASR gel formation, which might temporarily increase the strength slightly. As more ASR gel is formed and more moisture is absorbed by the gel, cracks form and a decrease in strength is observed. A careful examination of Figure 16b with consideration to the error bars reveals a general trend of decreasing strength with increasing NaOH addition up to and including the strength at 180 days. However, in general, a slightly lower strength (ranging from 4.5% to 1.4%) was observed compared to the strength at 28 days. This is attributed to the microstructural damage due to ASR reactions and the reduction in strength was found to be similar in magnitude to the reduction reported in literature [72]. The reason for the difference in the strengths of NaOH-8 specimens prepared in two separate batches, shown in Figure 16a and b, respectively, is potentially due to the better concrete quality in the smaller mixing volume size. Figure 16c shows the compressive strength of the cylinders from NaOH-00 and NaOH-12 in different environments. The cylinders from the NaOH-12 mixture in the environmental chamber showed a lower strength compared to the other cylinders. This difference was up to 25% compared to the cylinders from the NaOH-00 mixture conditioned outside. The compressive strength of the cylinders from each mixture in any environment did not significantly change over time. In some cases, such as for the cylinders from the NaOH-00 mixture conditioned outdoors, the compressive strength at 550 days was more than that at 28 days of age but the difference was less than 15%. Figure 17 shows the results of the elastic modulus for the cylinders from the NaOH-00 and NaOH-12 mixtures in different environments. Despite the difference between the results of different cylinders over time, there was no indication of the influence of ASR and alkali content on the elastic modulus of the cylinders.

**Figure 18:**
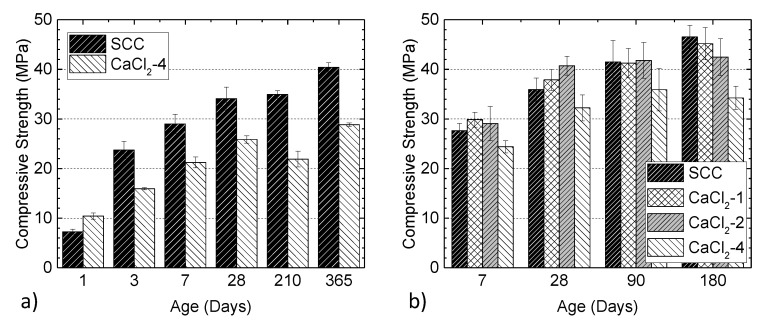
Effect of chloride ion on compressive strength at different ages: (**a**) SCC and CaCl_2_-4 mixtures from the larger batch series and (**b**) CaCl_2_ mixtures from the smaller batch series.

The effect of chloride ion on the compressive strength of the specimens from the larger batch series is shown in Figure 18a. By mixing 4% chloride, the day one compressive strength of concrete was increased by 44%. This is in agreement with the previous observations that the addition of calcium chloride accelerates the hydration rate of Portland cement concrete, resulting in a decrease in the setting time and an increase in the early strength of the pastes [75]. However, a lower strength was observed for the concretes in the presence of CaCl_2_ at later ages. Note that the slight drop in strength for the CaCl2-4 series at 210 days and then the increase at 365 days is attributed to the variation in the material properties. The strength due to tricalcium silicate (alite) and dicalcium silicate (belite) increased, but the strength due to tricalcium aluminate decreased with the addition of calcium chloride [76]. Additionally, calcium chloride retards the hydration of tricalcium aluminate while acting as accelerator for the hydration of the silicate phases [77]. The same trend was observed for the specimens with different levels of chloride (the smaller batch series), as shown in Figure 18b. It is seen that the strength of concrete at an early age increased with the addition of 1% and 2% chloride, while a 4% chloride addition resulted in a drop. At later ages, the strength gain of the mixture without CaCl_2_ was higher than the other mixtures. The overall strength of CaCl_2_-4 was the lowest among all mixtures. The reduction in the final strength might be attributed to the formation of chloroaluminate hydrates (a product of the reaction between chloride salt and the alumina phase (C_3_A) of cement), which is responsible for the concrete softening [75,78].

### 3.8. Split Tensile Strength

**Figure 19:**
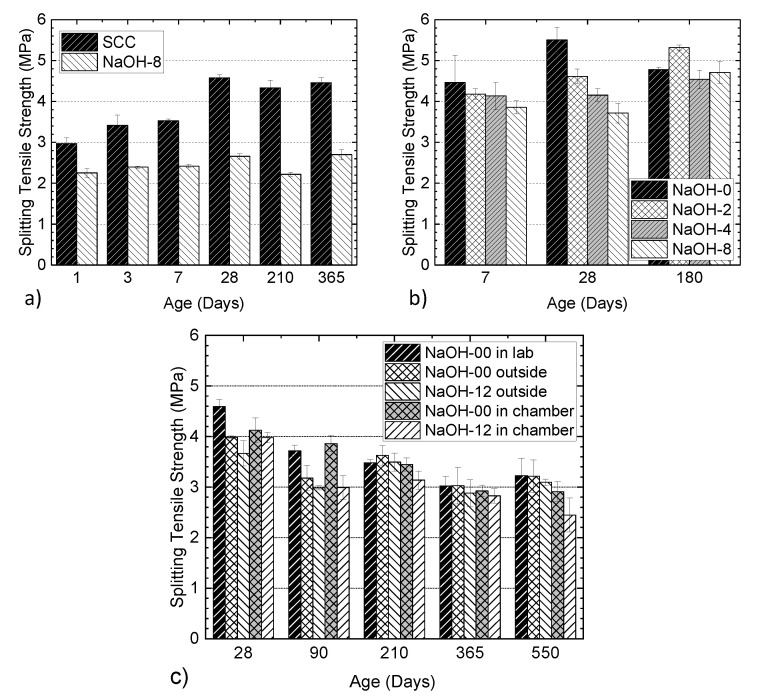
Influence of alkali addition on splitting tensile strength at different ages: (**a**) NaOH-8 and SCC mixtures from the larger batch series, (**b**) NaOH mixtures from the smaller batch series and (**c**) NaOH-00 and NaOH-12 mixtures.

The retarding effect of alkali on the strength development of concrete was also observed in the splitting tensile tests. For the specimens from the larger batch series, as shown in Figure 19a, the NaOH-8 mixture had less tensile strength than SCC (with the minimum difference being 24.2%). Again, these results are attributed to the more reticular and porous cement paste in the presence of alkali, the defects in concrete induced by ASR expansion in NaOH-8 samples and the pozzolanic reaction in the presence of fly ash in SCC, which not only increases the formation of C-S-H in the concrete but also mitigates ASR. As shown in Figure 19b, the observations were similar for split tensile strength to those of the compressive strength tests: the addition of NaOH results in a lower strength at an early age and a delayed strength development. At 180 days, a similar trend (decreasing strength with increasing NaOH content) to that at 28 days was expected, with the strength of each group being slightly lower compared to that of the same group at 28 days (i.e., similar to the compressive strength test results presented in Figure 16b).However, it is seen that the nonuniform ASR damage and the large variability in the brittle tensile failure have resulted in more scattered 180 days tensile strength. Figure 19c shows the split tensile strength of cylinders from NaOH-00 and NaOH-12 mixtures in different environments. At all ages after 90 days, the cylinders from NaOH-12 in the chamber showed a lower tensile strength. In addition to the increased (5.3 kg/m^3^) total alkali content, these cylinders were kept in an environment that accelerates ASR. The cylinders from this group showed about a 40% decrease in tensile strength at 550 days of age compared to that at 28 days. The cylinders from the NaOH-00 mixture kept in the laboratory showed a higher tensile strength at all ages compared to the other cylinders. At 28 days and 550 days of age, the cylinders from the NaOH-00 mixture in the lab had 13% and 25% higher tensile strength, respectively, compared to the cylinders from the NaOH-12 mixture in the environmental chamber. The cylinders from the NaOH-00 mixture kept in the laboratory showed a 30% reduction in the tensile strength at 550 days compared to the same at 28 days of age. It is concluded that ASR affected the tensile strength of the cylinders with reactive aggregates and normal doses of alkali provided by typical cements in the absence of supplementary cementitious materials such as fly ash.

### 3.9. Flexural Strength 

**Figure 20:**
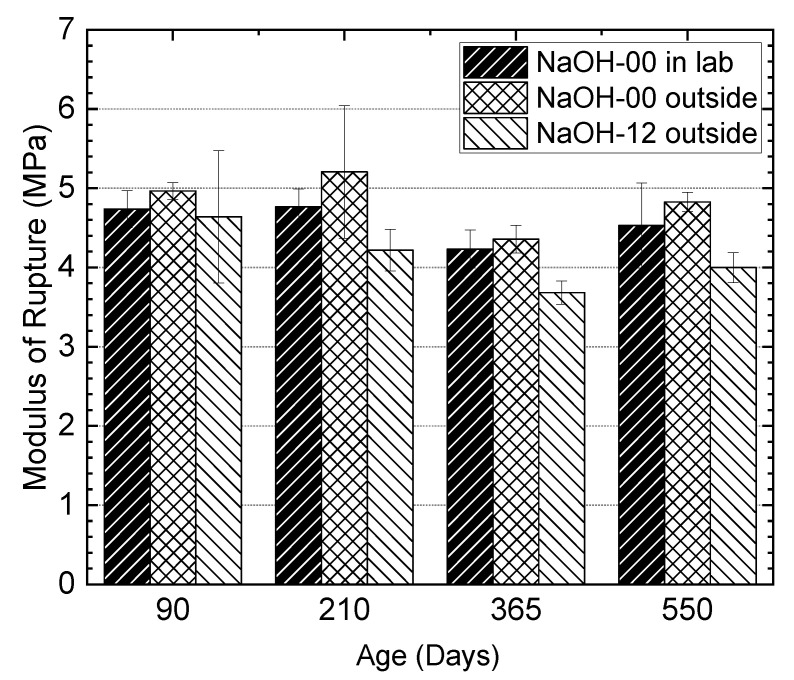
Effect of alkali addition on the modulus of rupture of concretes at different ages.

Figure 20 shows the results of the modulus of rupture for the prisms from the NaOH-00 and NaOH-12 mixtures conditioned in the laboratory and outside. At all ages, the prisms from the NaOH-12 mixture conditioned outside showed a lower modulus of rupture compared to the prisms from the NaOH-00 mixture conditioned in the laboratory or outside. However, the influence of ASR on the modulus of rupture at different ages was less than its influence on the tensile strength, as presented in Section 3.8. The maximum difference in the modulus of rupture between the prisms from NaOH-12 conditioned outside and the prisms from NaOH-00 at different ages was about 20%. The decline in the modulus of rupture of the prisms from NaOH-12 at 550 days compared to 28 days of age was about 15%.

## 4. Conclusions

This study reports on the results of two separate accelerated aging methods achieved through direct additions of sodium hydroxide and calcium chloride for ASR and rebar corrosion, respectively, and their impact on the properties of concrete. The following conclusions were drawn from the findings of this research:

(1) Using reactive fine aggregate, no fly ash and adding alkalis in the form of NaOH directly into the fresh concrete paste was found to be an effective method to accelerate ASR of specimens kept in ambient outdoor conditions. The increase in total alkali loading resulted in a decrease in the compressive and splitting tensile strength of concrete at 28 days but an increase in strength after 180 days. When the error bars are considered, the general trend of decreasing strength with increasing NaOH addition is observed, suggesting accelerated ASR in the presence of alkali.
(2) The first ASR cracks on specimens with a total alkali loading of 5.57 kg/m^3^ were observed at around 90 days and the crack intensity increased over time during the 432-day measurement period. While the crack intensity on the surface increased and the cracks coalesced, the crack widths remained between 0.05 mm and 0.1 mm throughout the measurement period of 432 days.
(3) The addition of chloride accelerated the rebar corrosion, evidenced by the reduced open circuit corrosion potential measurements and crack maps. Cracks due to corrosion in specimens with 4% chloride by weight of cement were first observed approximately 90 days after casting. Differently from the cracks generated by ASR, both the crack length and width increased with time.
(4) The effect of 4% chloride addition on the physical and mechanical properties of concrete was observed to be more severe than that of 1% and 2% chloride, indicating that the corrosion of the embedded steel rebar with a high probability occurred at a chloride level of 4%.
(5) The bulk diffusivity results show that the chloride diffusivity of concrete with fly ash and no NaOH addition was lower than that of the mix without fly ash and a 0.8% NaOH addition. This indicates the role of sodium hydroxide in increasing the porosity of the ensuing concrete in addition to the increased diffusivity created from the interconnectedness of micro-cracks due to ASR gel expansion.
(6) In comparison with SCC, a lower surface resistivity was observed for the NaOH-8 mixture and its difference from the SCC grew over time. The addition of chloride resulted in a decreased surface resistivity and the chloride permeability of concrete increased with increasing chloride addition.
(7) The concrete reinforcement ratio was shown to reduce the ASR expansion. Increasing the longitudinal reinforcement ratio of concrete from 0% to 3.41% reduced the ASR-induced expansion by 29% to 56% in different environments. The ASR expansion of plain concrete prisms from the mixture with a 5.3 kg/m^3^ total alkali content was 0.62% during the 575 days in the outside environment, while this value for the same concrete with a 3.41% reinforcement ratio was 0.28%.

In summary, this study not only addresses an existing research gap on the influence of the addition of chemicals for accelerated aging in concrete on the ensuing concrete properties, but it also covers a range of variables that influence ASR and reinforcement corrosion. The dosage of the chemicals for accelerated aging, concrete ingredients, batch size, sample size, reinforcement ratio and curing conditions were among the variables that were studied. The degradation of the physical and mechanical properties of concrete due to aging were assessed over a wide range of variables and time. The generated datasets helped create a more complete picture of the range of effects from ASR and corrosion. Additionally, the ASR expansion saturation over time towards different limits based on the reinforcement ratio were defined as an estimate of potential effects in RC structures with varying reinforcement detailing. It is noted that the results of this research are influenced by experimental uncertainty. The application of these results to any engineering design/verification can be affected by this uncertainty, in addition to other aleatory and epistemic uncertainties.

## Figures and Tables

**Table 1:** Chemical composition of cements and fly ash.

Chemical Compositions (wt.%)				Mineralogical Phase Composition of Cement (wt.%)		
Materials	Fly Ash	Cement	Cement			
Type/Class	F	I/II	II	Type	I/II	II
SiO_2_	58.21	21.11	20.9	Tricalcium silicate (C_3_S)	58	55
Al_2_O_3_	24.06	4.43	4.0	Dicalcium silicate (C_2_S)	17	18
Fe_2_O_3_	4.13	3.87	3.6	Tricalcium aluminate (C_3_A)	5	5
CaO	8.12	63.96	63.9	**Na_2_O equivalent (wt.%)**		
MgO	1.96	1.54	1.9	**Fly ash**	**Cement**	
				**Class F**	**Type I/II**	**Type II**
SO_3_	0.29	2.48	2.8	0.84	0.49	0.58

**Table 2:** Proportions of the concrete mixtures.

Concrete Mixtures	Materials (kg/m^3^)									Admixtures (mL/m^3^)			
	Cement Type		Fly Ash	Sand	Pea Gravel	Crushed Rock	Water	NaOH	CaCl_2_	1 ^a^	2 ^b^	3 ^c^	4 ^d^
	I/II	II											
NaOH-0	432	–	–	1087 ^e^	1228 ^f^	–	213	–	–	1155	2320	–	–
NaOH-00	–	291	–	727 ^g^	208 ^h^	1038	154	3.6	–	–	–	1238	1625
NaOH-2	432	–	–	1087 ^e^	1228 ^f^	–	213	0.9	–	1155	2320	–	–
NaOH-4	432	–	–	1087 ^e^	1228 ^f^	–	213	1.8	–	1155	2320	–	–
NaOH-8	432	–	–	1087 ^e^	1228 ^f^	–	213	3.7	–	1155	2320	–	–
NaOH-12	–	291	–	727 ^g^	208 ^h^	1038	154	3.6	–	–	–	1238	1625
SCC	324	–	108	1087 ^e^	1228 ^f^	–	213	–	–	1155	2320	–	–
CaCl_2_-1	324	–	108	1087 ^e^	1228 ^f^	–	213	–	4.9	1155	2320	–	–
CaCl_2_-2	324	–	108	1087 ^e^	1228 ^f^	–	213	–	9.8	1155	2320	–	–
CaCl_2_-4	324	–	108	1087 ^e^	1228 ^f^	–	213	–	19.7	1155	2320	–	–

^a^ Admix 1: Pozzolith 200N; ^b^ Admix 2: Glenium 7500N; ^c^ Admix 3: Pozzolith 8; ^d^ Admix 4: Glenium 3400NV; ^e^ Brazos River Sand; ^f^ Pea Gravel I; ^g^ El Paso Sand; ^h^ Pea Gravel II.

**Table 3:** Test plan for the series of larger batches of concrete mixtures with Type I/II cement and Brazos reactive sand: SCC, NaOH-8 and CaCl_2_-4.

Standards	ASTM C39 [53], ASTM C496 [54], ASTM G1-03 [55], NT-build 208 [56], NT-build 443 [57], ASTM C876 [58], AASHTO TP 95-14 [59], ASTM D4262 [60]
Specimen type	Cylinders for compressive and split tensile strength (100 × 200 mm)
	Chloride penetration specimens (for SCC and NaOH-8 mixes only) (100 × 100 mm)
	Cylindrical specimen for mass loss and open circuit corrosion potential measurements: 75 × 254 mm cylinder with a single embedded #10 rebar at the center
Property measured ^a^	Compressive strength, splitting tensile strength, mass loss, chloride content, chloride penetration, open circuit corrosion potential, surface resistivity, pH
Test ages	Compressive and splitting tensile strength: 1 day, 3 days, 7 days, 28 days, 210 days and 365 days Chloride penetration: 28 days, 180 days and 365 days

^a^ The testing plan for all three mixtures is identical except that chloride penetration was measured only on SCC and NaOH-8 mixtures.

**Table 4:** Test plan for the series of smaller batches of concrete mixtures with Type I/II cement and Brazos reactive sand containing different levels of NaOH and CaCl_2_.

	NaOH ^a^	CaCl_2_^b^
Standards	ASTM C39 [53], ASTM C496 [54], ASTM C642 [61], AASHTO TP 95-14 [59]	ASTM C39 [53], ASTM C496 [54], AASHTO TP 95-14 [59], ASTM C876 [58]
Specimen type	Cylinders for compressive and split tensile strength (100 × 200 mm)	
	Cylinders for density, absorption and void (75 × 150 mm)	
	Unnotched prisms for length change measurement (100 × 100 × 350 mm)	Cylindrical specimen for open circuit corrosion potential measurements 75 × 254 mm cylinders with embedded #10 bar (9.525 mm) in the middle
Property measured	Compressive strength, splitting tensile strength, density, absorption and void, surface resistivity	Compressive strength, splitting tensile strength, density, surface resistivity, open circuit corrosion potential
Test ages	Density, absorption and void: 28 days Compressive and split tensile strength: 7 days, 28 days, 90 days and 180 days	

^a^ The testing plan for NaOH-0 specimens is identical to other NaOH mixtures; ^b^ The testing plan for SCC specimens is identical to other CaCl_2_ mixtures.

**Table 5:** Test plan for the concrete mixtures with Type II cement and El Paso sand: NaOH-00 and NaOH-12.

Standards	ASTM C39 [53], ASTM C496 [54], ASTM C78 [62]
Specimen type	Cylinders for compressive and split tensile strength (100 × 200 mm)
	Prisms for flexural strength (150 × 150 × 525 mm)
	Prisms for expansion (150 × 150 × 525 mm; 0%, 1.23%, 2.18% and 3.41% reinforcement ratios)
Property measured	Compressive strength, splitting tensile strength, elastic modulus, modulus of rupture, expansion
Test ages	Compressive and splitting tensile strength: 28 days, 90 days, 210 days, 365 days, 550 days Flexural strength: 90 days, 210 days, 365 days Expansion: once every other week for 575 days

**Table 6:** Density, void and absorption of concretes at 28 days.

Mix	Bulk Density, Dry (g/cm^3^)	Bulk Density after Immersion (g/cm^3^)	Apparent Density (g/cm^3^)	Absorption after Immersion (%)	Absorption after Immersion and Boiling (%)	Void (%)
NaOH-0	1.66	1.74	1.81	4.62	4.81	8.00
NaOH-2	1.64	1.72	1.78	4.48	4.65	7.64
NaOH-4	1.64	1.71	1.77	4.22	4.44	7.28
NaOH-8	1.65	1.73	1.79	4.65	4.90	8.09
SCC	1.67	1.75	1.82	4.44	4.86	8.13
CaCl_2_-1	1.65	1.73	1.80	4.70	5.02	N.A. ^a^
CaCl_2_-2	1.65	1.73	1.79	4.49	4.83	N.A.
CaCl_2_-4	1.66	1.74	1.80	4.58	4.86	N.A.

^a^ N.A.: Not available.

**Table 7:** Average effective chloride transport coefficient, D_e_, for the larger batch series of SCC and NaOH-8 specimens.

Mix	28 Days	210 Days	365 Days
	D_e_ (10^−11^ m^2^/s)	D_e_ (10^−11^ m^2^/s)	D_e_ (10^−11^ m^2^/s)
SCC	0.964	1.18	1.10
NaOH-8	4.86	5.09	7.27

## Abbreviations

ASR	alkali–silica reactivity
DEMEC	demountable mechanical strain gauge
RC	reinforced concrete
RH	relative humidity
SCC	self-consolidating concrete
